# Comparison of Raman spectroscopy with mass spectrometry for sequence typing of *Acinetobacter baumannii* strains: a single-center study

**DOI:** 10.1128/spectrum.01425-24

**Published:** 2025-02-05

**Authors:** Suling Liu, Ni Zhang, Jiawei Tang, Chong Chen, Weisha Wang, Jingfang Zhou, Long Ye, Xiaoli Chen, ZhengKang Li, Liang Wang

**Affiliations:** 1 Department of Laboratory Medicine, Guangdong Provincial People’s Hospital (Guangdong Academy of Medical Sciences), Southern Medical University, Guangzhou, Guangdong, China; 2 School of Medical Informatics and Engineering, Xuzhou Medical University 499782 , Xuzhou, Jiangsu Province, China; 3 Joint International Research Laboratory of Agriculture and Agri-Product Safety, Ministry of Education of China, Institutes of Agricultural Science and Technology Development, Yangzhou University, Yangzhou, Jiangsu, China; University of Guelph College of Biological Science, Guelph, Canada

**Keywords:** *Acinetobacter baumannii*, surface-enhanced Raman spectroscopy, machine learning, multi-locus sequence typing, MALDI-TOF

## Abstract

The rapid sequence typing (ST) of bacterial strains is crucial for effective nosocomial infection control and mitigating the spread of nosocomial pathogens, e.g., *Acinetobacter baumannii*. While accurate in identifying *A. baumannii* strains, current typing methods are often impractical in clinical settings due to their time-consuming nature. This study developed a novel approach, combining surface-enhanced Raman spectroscopy (SERS) with machine-learning (ML) algorithms, to construct predictive models for *A. baumannii* sequence typing based on SERS spectra. The objective was to assess the feasibility of this integrated method for efficient sequence typing of *A. baumannii* strains. Clinically isolated *A. baumannii* strains (*N* = 267) were collected from a single hospital between 2013 and 2023. Based on multilocus sequence typing, 39 STs of *A. baumannii* were identified. Then, a SERS spectral database for all these strains was constructed, and predictive models based on eight ML algorithms were developed to predict SERS signals to determine their STs, among which the support vector machine (SVM) model had the best performance (fivefold cross-validation = 99.74%). The typing capacity of the SERS-SVM method was compared with that of matrix-assisted laser desorption/ionization time-of-flight (MALDI-TOF) for *A. baumannii* sequence typing, confirming the superiority of SERS-SVM over MALDI-TOF mass spectrometer. This pilot study lays the groundwork for employing the SERS-ML method to rapidly identify *A. baumannii* strain types in clinical laboratories, aiding in controlling bacterial pathogen transmission. Further studies are warranted to evaluate its potential in nosocomial surveillance systems, especially for rapidly identifying outbreaks within hospitals.

**IMPORTANCE:**

The rapid and accurate sequence typing (ST) of bacterial pathogens is pivotal in controlling transmission within healthcare settings. *Acinetobacter baumannii* infection, known for its high transmissibility and drug resistance, presents a major challenge in nosocomial infection control. In this study, surface-enhanced Raman spectroscopy (SERS) was used to differentiate *A. baumannii* strains with distinct STs based on unique Raman spectral profiles. We then constructed and compared eight machine-learning models on SERS spectra to quickly identify bacterial STs. The results showed that the support vector machine model outperformed matrix-assisted laser desorption/ionization time-of-flight mass spectrometer in determining *A. baumannii* STs. This approach enables rapid identification of *A. baumannii* variants with different STs, supporting the early detection and control of nosocomial infections by this multidrug-resistant pathogen.

## INTRODUCTION

The emergence of multidrug-resistant, hospital-acquired infections caused by *Acinetobacter baumannii* has become a global concern (1–3). Depending on the bacterial sequence types (STs), mortality rates range from 8% to 35%, leading to increased hospitalization time and healthcare expenditures (4, 5). The nosocomial spread of multidrug-resistant *A. baumannii* strains, which are challenging to eradicate, is particularly concerning (6, 7). A single-center study in Mexico revealed an unusual case that led to nosocomial transmission of *A. baumannii* between 2011 and 2015, resulting in 53 deaths within 30 days of infection (8). Currently, tracking nosocomial infections caused by *A. baumannii* and its transmission primarily relies on the multilocus sequence typing (MLST)-based method (9, 10), which leads to improved prevention and control of the bacterial pathogen. However, the prevalent identification methods, primarily based on DNA analysis, including MLST, amplified fragment length polymorphism, microsatellite typing, whole-genome sequencing, and pulsed-field gel electrophoresis are costly and time-consuming (11–13). These limitations often result in delayed intervention and inadequate control of nosocomial infections in hospital settings, preventing patients from efficient and effective therapeutical needs. Consequently, there is an urgent need for rapid and direct methods to identify *A. baumannii* STs.

Matrix-assisted laser desorption/ionization coupled with time-of-flight mass spectrometry (MALDI-TOF MS) is well recognized for single microbial identification and widely used in clinical fields due to its specificity, speed of analysis, and low cost of consumables. Recently, MALDI-TOF MS has made some progress in drug resistance and sequence typing. Through the analysis software and clustering algorithm provided by the manufacturer, the spectra of the same strains were analyzed and compared, and the homology and heterogeneity were measured by the distance, which could be used for hospital traceability (14, 15), making it a potential tool for typing bacterial strains. On the other hand, surface-enhanced Raman spectroscopy (SERS) is widely employed for identifying and characterizing microorganisms and is known for its high accuracy and rapidity (16, 17). Recently, an increasing number of studies have highlighted that SERS coupled with machine learning (ML) represents a notably simpler, faster, and more effective method for microbial identification (18, 19). Clinical studies have demonstrated enhanced species identification of *Staphylococci* and carbapenem-resistant and sensitive *Klebsiella pneumoniae* strains using this approach (20, 21). However, research on using SERS-ML in identifying the sequence types of *A. baumannii* remains limited and is worth exploring.

In this study, we investigated the utility of MALDI-TOF MS and the SERS-ML method for typing *A. baumannii* strains. Initially, the study utilized MLST to classify all the *A. baumannii* strains (*N* = 267) isolated from clinical samples. These strains were then analyzed for clustering analysis using MALDI-TOF MS spectral data. Meanwhile, a comprehensive collection of *A. baumannii* Raman spectral profiles via SERS was collected. To construct predictive models, machine-learning algorithms were optimized to extract generic and ST-specific characteristic peaks. Finally, eight ML models were constructed and compared to find the optimal predictive model. Taken together, this novel approach aims to expedite the identification of *A. baumannii* strain types, thereby enhancing nosocomial bacterial infection control and prevention.

## MATERIALS AND METHODS

### *A. baumannii* strains and sequence typing

The strains were isolated directly from clinical samples and cultured on Columbia blood agar plates at 35°C for 18–24 hours in the Department of Laboratory Medicine, Guangdong Provincial People’s Hospital (Guangdong Academy of Medical Sciences), Southern Medical University, Guangzhou, China. The sequence types of *A. baumannii* strains were established using MLST as a standard reference. The contig sequences from assembled genomes were aligned with the alleles of seven housekeeping genes (*cpn60*, *fusA*, *gltA*, *pyrG*, *recA*, *rplB*, and *rpoB*) from the pubMLST database using BLASTN (version: ncbi-blast-2.9.0+; parameters: -evalue 1e-5, -outfmt 6, -num_alignments 10000). Exact matches for each gene determined the ST by comparing the allele profiles. The strains were preserved at −80°C for future testing.

### MALDI-TOF MS and clustering analysis

First, the strains were subcultured onto blood agar plates and incubated at 35°C for 18–24 hours. Then, VITEK MS-Target (3 × 16 + 3c) target plates were coated, and 1 µL of CHCA matrix was added. Next, detection was performed using the VITEK MS RUO system to collect protein spectra in the 2,000–20,000 Da range. We conducted a clustering analysis of these protein spectra using BioNumerics software. During the data analysis, the Kruskal-Wallis test was used to identify differences in mass spectral peaks between MLST clusters, and the Pearson correlation coefficient was calculated to assess the similarity between strains. Subsequently, an unweighted pair group method with arithmetic mean clustering tree was constructed based on the differential peaks among MLST clusters, and the clustering effectiveness was evaluated using the cophenetic correlation coefficient.

### Preparation of silver nanoparticles

The SERS technique is employed to amplify the Raman spectral signal, thereby reducing errors due to weak signals. Silver nanoparticles (AgNPs) were utilized as enhancers, following a previously described preparation procedure. Initially, 33.72 mg of silver nitrate (AgNO_3_) was added to a triangular flask containing 200 mL of deionized distilled water (ddH_2_O). The mixture was stirred and heated to boiling. Then, 8 mL of sodium citrate (Na_3_C_6_H_5_O_7_) was added while stirring and heating at a rate of 650 revolutions/minute for 40 minutes. After the heat treatment was discontinued, the mixture was stirred until it reached room temperature. The volume of the solution was subsequently adjusted to a final volume of 200 mL with ddH_2_O. Subsequently, 1 mL of this solution was transferred to a clean Eppendorf (EP) tube and centrifuged at 7,000 revolutions/minute for 7 minutes. The supernatant was discarded, and the precipitate was resuspended in 100 µL of ddH_2_O. The AgNP substrate was stored at room temperature in the dark for long-term storage.

### SERS spectral acquisition

Each bacterial strain was recovered by cultivation on an agar plate overnight. A single colony from each species was then inoculated into 15 µL of phosphate-buffered saline, mixed thoroughly via vortexing, and combined with 15 µL of negatively charged AgNP substrate solution. The resulting suspension was carefully deposited onto a silicon wafer to form a circular spot. After natural drying, SERS was performed on the sample. Raman spectral analysis was conducted using the Anton Paar Cora^100^ hand-held Raman spectrometer (Anton Paar Shanghai Trading Co., Ltd., China), configured with the following parameters: (i) excitation wavelength: 784.56 nm, (ii) excitation power: 25 mW, (iii) spectral resolution: 1 nm, (iv) spectral wave number resolution: 10 cm^−1^, and (v) detection spectral range: 400–2,300 cm^−1^. The SERS spectra were calibrated using the Raman peak at 520 cm^−1^ as the reference peak, with integration time accounting for the dark current deduction. For each bacterial species, 200 spectra were collected by randomly selecting detection sites within each dried sample spot.

### Data preprocessing of SERS spectra

In Raman spectral analysis, raw data preprocessing is necessary to enhance the signal-to-noise ratio and normalize the spectral distribution. This process involves curve smoothing, denoising, baseline correction, and spectral normalization. Average Raman spectra were generated for each strain by averaging the calculated intensities at each Raman shift from 519.56 cm^−1^ to 1,800.18 cm^−1^. These average Raman spectra were then processed and smoothed using LabSpec 6 (Horiba Scientific, Japan). Characteristic peaks were determined through the following steps: (i) the “Smoothing” function was applied to the average Raman spectrum (parameters: degree = 4, size = 5, height = 50). (ii) Baseline correction was performed using a polynomial function (parameters: type = polynom, degree = 6, attach = NO), with the “Auto” option for characteristic peak determination. (iii) Auto-normalization of spectral data was performed using LabSpec 6 to compare Carbapenem-sensitive Klebsiella pneumoniae (CSKP) and Carbapenem-resistant Klebsiella pneumoniae (CRKP) curves. (iv) Identification of characteristic peaks with the GaussLoren function (parameters: level = 0%, size = 32, default settings for other parameters). All the characteristic peaks are marked with black arrows. Error bands were generated for the averaged Raman spectra of CSKP and CRKP using Origin software. These error bands, based on a 20% standard deviation of the Raman effect strength at each Raman shift, indicate the experiment’s reproducibility. Mean Raman spectra and 20% standard error bands were also generated for each *A. baumannii* strain, including their characteristic peaks.

### Deconvolution and barcoding

Deconvolution was applied to the SERS spectra of each strain to explore the distinct characteristics of average Raman spectra for various *A. baumannii* STs. To enhance the accessibility of the spectral data, barcodes representing the deconvoluted Raman spectra were created, categorizing the spectra based on different variants and subtypes (22). The detailed approach to deconvolution and barcoding has been described previously (23).

### Dimensionality reduction and clustering visualization

To discern the intrinsic differences among the SERS spectra of various *A. baumannii* STs, orthogonal partial least squares discriminant analysis (OPLS-DA), principal component analysis (PCA), and *t*-distributed stochastic neighbor embedding (TSNE) were utilized for dimensionality reduction and clustering visualization. We selected the first two feature dimensions after TSNE dimensionality reduction as the coordinate axes for data visualization and presented the *A. baumannii* categories as scatter plots. Additionally, SIMCA software (Umetrics, Sweden) was used to process the raw data to enable intuitive differentiation of SERS spectra among different STs of *A. baumannii*. Different spectral data are represented using distinct colors, with dashed circles and labels indicating the corresponding categories. Please refer to our previously published studies for a comprehensive and detailed algorithmic analysis and annotation of the SERS spectra (23).

### Comparison and evaluation of machine-learning models

Eight machine-learning algorithms were employed to differentiate STs, namely, Adaptive Boosting (AdaBoost), Bagging, Decision Tree (DT), eXtreme Gradient Boosting (XGBoost), Gradient Boosting (GBoost), Quadratic Discriminant Analysis (QDA), Random Forest (RF), and support vector machine (SVM). The SERS matrix was divided into training, validation, and test sets in a 6:2:2 ratio using train_test_split before inputting different ML algorithms for model construction and performance validation. Additionally, each ML model undergoes GridsearchCV to identify the optimal hyperparameter combinations, ensuring adequate model fitting. Model performance was evaluated using four metrics: accuracy, precision, recall, and F1 score, along with three evaluation methods: fivefold cross-validation (CV), receiver operating characteristic (ROC) curves, and confusion matrices. All algorithms are executed based on SciKit-Learn (version 0.21.3) and PyCharm (version 2022.2.5).

## RESULTS

### Technology roadmap and standardized database

**Fig 1:**
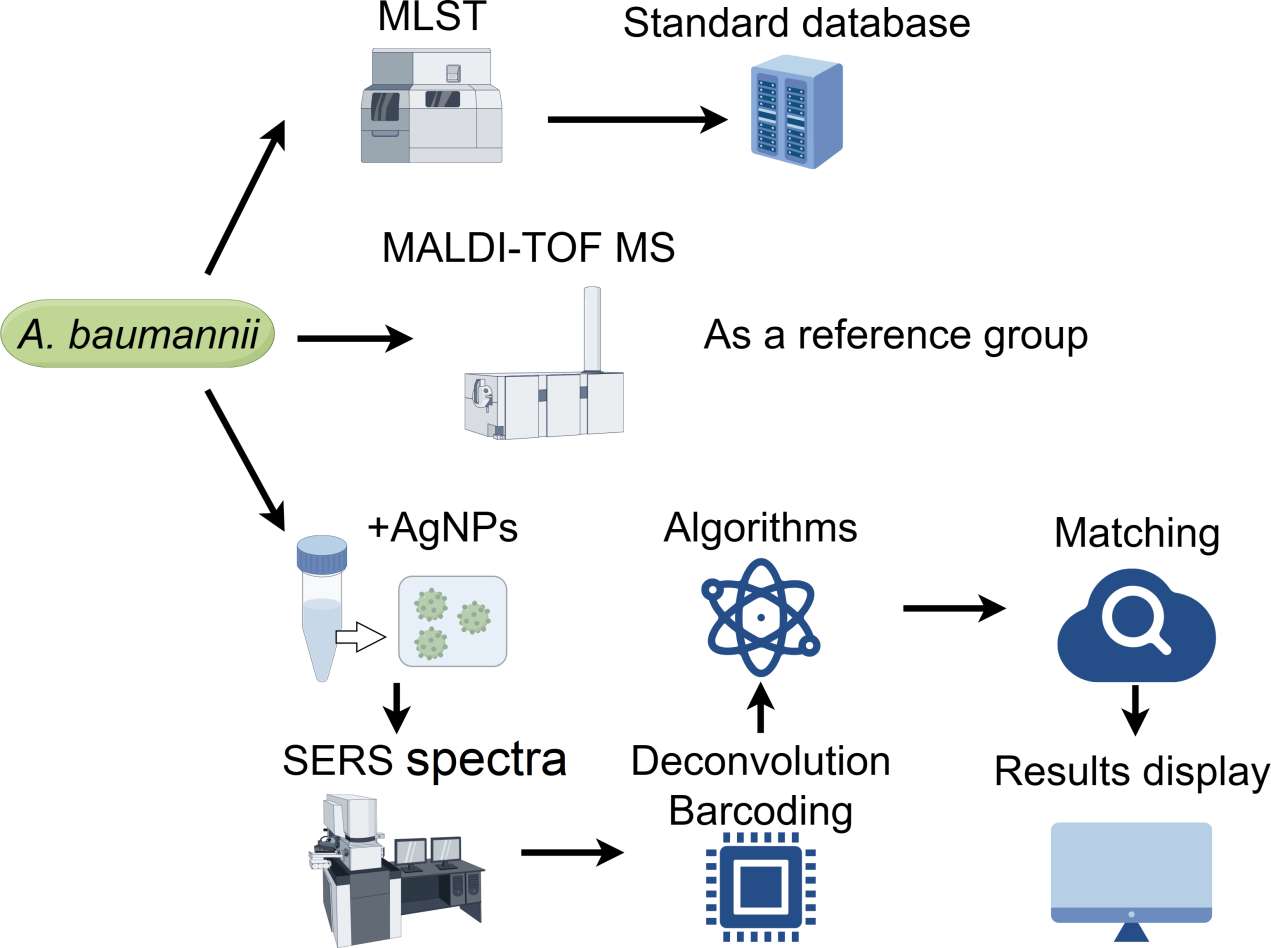
The schematic illustration of the study roadmap. The MLST method was employed to establish a standard database. MALDI-TOF MS was utilized as a control reference group. The SERS spectral data were then acquired and processed to construct predictive machine-learning models for *A. baumannii* strain typing. This figure was created with FigDraw and is licensed under copyright to Dr. Suling Liu.

All the *A. baumannii* strains (*N* = 267) were isolated from clinical samples. Subsequently, all STs were identified using the MLST method as the standard reference for identifying bacterial strains. This process led to the generation of a standardized database (Fig. 1). In addition, these STs were identified using MALDI-TOF MS. Next, the Raman spectral signals were enhanced with the AgNPs, and SERS spectra were captured. Machine-learning algorithms were employed to analyze, recognize, and classify the collected SERS spectra of all *A. baumannii* strains.

### Limitation of MALDI-TOF MS for *A. baumannii* strain typing

**Fig 2:**
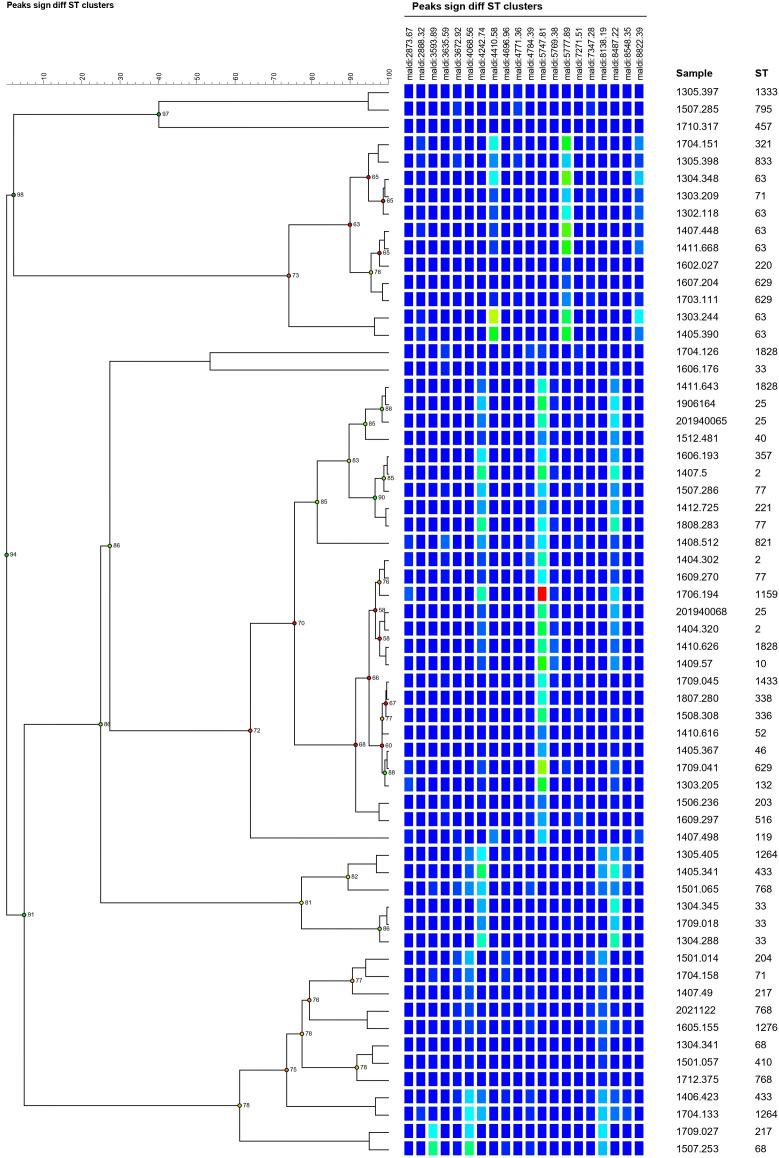
MALDI-TOF MS was employed as a comparative method for identifying all *A. baumannii* strains. The peak intensities were visually represented using a color scheme ranging from low to high intensity in the sequence of blue, green, yellow, and red. As a result, these strains were successfully identified, exhibiting variations in certain peak intensities.

The MLST method, currently regarded as the reference, was initially utilized to identify the STs of the collected strains (Table 1Table 1). This process identified 39 distinct STs of *A. baumannii*, forming the basis of the study’s standard database. The results obtained from other identification methods were subsequently compared with those from the standard database to verify the accuracy of the proposed method. The strains were then typed via the MALDI-TOF MS method, a common tool employed in hospital laboratories for bacterial identification. The dendrogram depicted in Fig. 2, derived from MALDI-TOF MS, revealed the distinct clustering patterns of *A. baumannii* strains based on the unique MS spectral profiles. However, some of the strains showed indistinguishable patterns due to the similar MS peak intensities. This observation underscores the necessity of more effective methods for identifying *A. baumannii* STs accurately.

**TABLE 1:** The STs of all the *A. baumannii* strains included in this study

Strains	ST	*cpn60*	*fusA*	*gltA*	*pyrG*	*recA*	*rplB*	*rpoB*
1404.302	2	2	2	2	2	2	2	2
1404.32	2	2	2	2	2	2	2	2
1407.5	2	2	2	2	2	2	2	2
1409.57	10	1	3	2	1	4	4	4
1607.211	25	3	3	2	4	7	2	4
1906164	25	3	3	2	4	7	2	4
201940065	25	3	3	2	4	7	2	4
201940068	25	3	3	2	4	7	2	4
1304.288	33	3	5	7	1	7	1	4
1304.345	33	3	5	7	1	7	1	4
1606.176	33	3	5	7	1	7	1	4
1709.018	33	3	5	7	1	7	1	4
1512.481	40	1	2	2	2	5	1	14
1405.367	46	5	12	11	2	14	9	14
1410.616	52	3	2	2	7	9	1	5
1302.118	63	17	20	23	10	20	13	20
1303.244	63	17	20	23	10	20	13	20
1304.348	63	17	20	23	10	20	13	20
1405.39	63	17	20	23	10	20	13	20
1407.448	63	17	20	23	10	20	13	20
1411.668	63	17	20	23	10	20	13	20
1408.542	64	17	21	23	10	20	13	20
1508.319	64	17	21	23	10	20	13	20
1605.125	64	17	21	23	10	20	13	20
1609.271	64	17	21	23	10	20	13	20
20204032	64	17	21	23	10	20	13	20
1304.341	68	20	24	26	14	23	16	23
1507.253	68	20	24	26	14	23	16	23
1303.209	71	20	26	26	14	26	16	25
1704.158	71	20	26	26	14	26	16	25
1507.286	77	3	2	2	2	3	4	28
1609.27	77	3	2	2	2	3	4	28
1808.283	77	3	2	2	2	3	4	28
1509.365	106	3	3	16	1	13	1	1
1407.498	119	36	20	38	16	38	18	20
1303.205	132	3	5	5	1	7	1	4
1506.236	203	3	4	2	2	7	1	2
1501.014	204	22	26	47	18	27	16	47
1407.49	217	20	24	26	14	23	19	23
1709.027	217	20	24	26	14	23	19	23
1602.027	220	45	20	44	16	20	29	20
1412.725	221	3	1	2	1	18	1	48
1704.151	321	45	58	44	10	20	18	56
1508.308	336	27	2	2	2	7	2	5
1807.28	338	8	5	5	26	13	1	2
1606.193	357	3	4	2	2	7	2	5
1702.049	396	60	21	46	10	20	18	20
1501.057	410	20	26	26	14	26	16	23
1405.341	433	22	26	29	14	27	16	47
1406.423	433	22	26	29	14	27	16	47
1710.317	457	66	20	48	35	72	18	62
1609.297	516	3	4	2	2	9	1	2
1607.204	629	98	20	46	16	20	18	62
1703.111	629	98	20	46	16	20	18	62
1709.041	629	98	20	46	16	20	18	62
1501.065	768	22	26	91	18	23	19	47
1712.375	768	22	26	91	18	23	19	47
2021122	768	22	26	91	18	23	19	47
1507.285	795	101	20	46	10	129	18	116
1408.512	821	12	100	2	2	9	1	52
1305.398	833	45	58	44	10	20	18	116
1706.194	1159	1	2	2	2	5	4	14
1305.405	1264	22	26	90	14	23	16	49
1704.133	1264	22	26	90	14	23	16	49
1605.155	1276	192	26	91	14	192	16	47
1305.397	1333	106	20	119	65	144	36	111
1709.045	1433	3	1	5	3	6	2	3
1410.626	1828	3	2	2	2	7	2	2
1411.643	1828	3	2	2	2	7	2	2
1704.126	1828	3	2	2	2	7	2	2

### Characteristic peaks of SERS spectra

**Fig 3:**
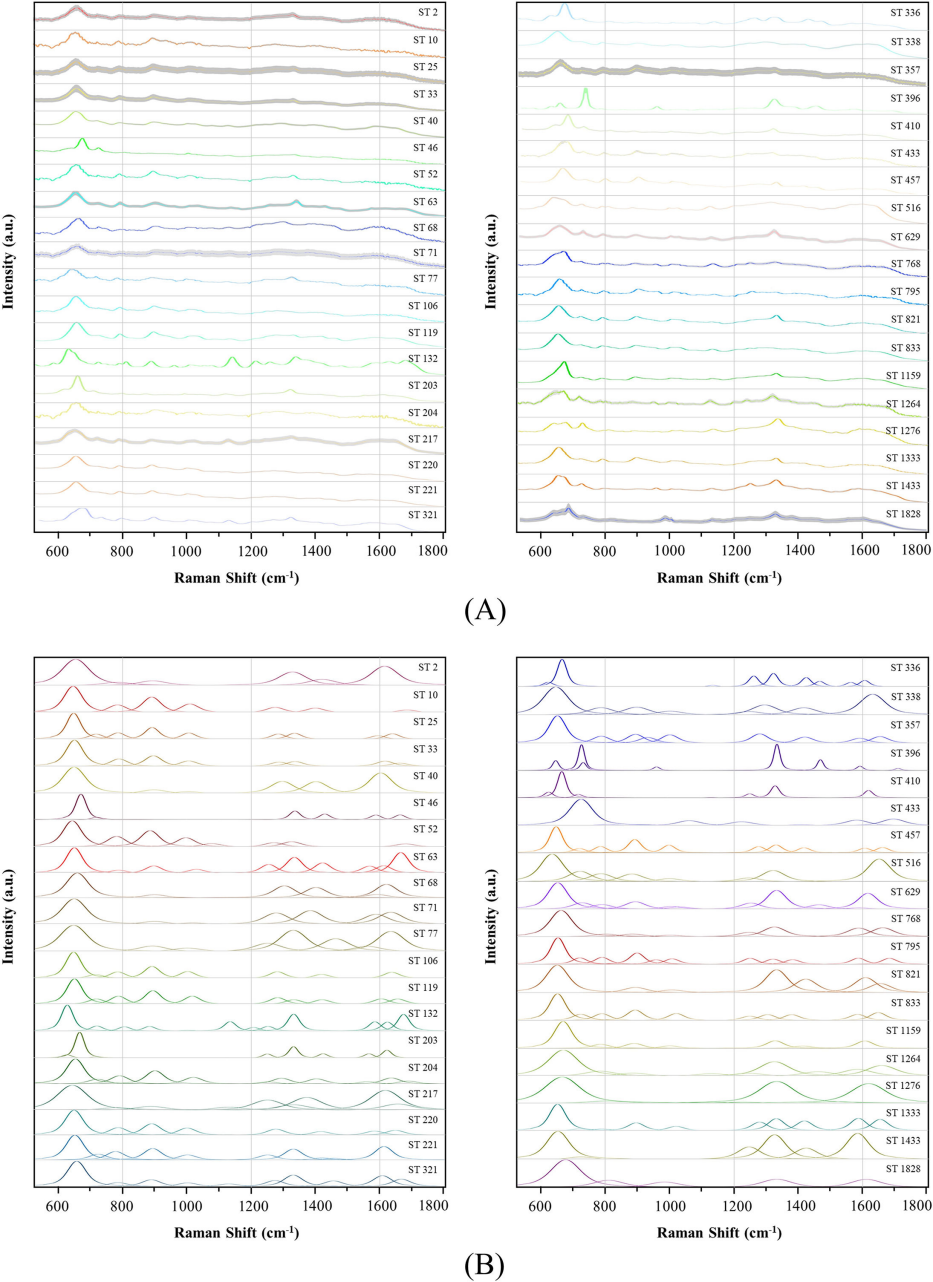
The visualization of SERS spectra for *A. baumannii* strains. (**A**) The average spectra of 39 STs of *A. baumannii*, with the shaded area representing the standard error band, highlight the quality of the data. (**B**) The deconvolution spectra of the 39 STs were generated to amplify the differences between the average SERS spectra.

After constructing a comprehensive database of characteristic peaks specific to *A. baumannii*, the resulting average spectra of 39 STs were obtained (Fig. 3A). Subsequent deconvolution processing of these spectra generated corresponding deconvolution graphs (Fig. 3B), amplifying the discrepancies among different STs. Although most of the peaks within these graphs exhibited slight variations, only a select few displayed noteworthy differences critical for ST differentiation. In addition, these distinctive characteristic peaks were appropriately labeled, and their spectral intensities were visually represented in dot plots (Fig. S1). The dot plot displays the combinations and intensity distributions of different STs of characteristic peaks. Comparing the differences between these characteristic peaks can aid in the accurate identification of specific STs.

### Clustering analysis of *A. baumannii* SERS spectra

**Fig 4:**
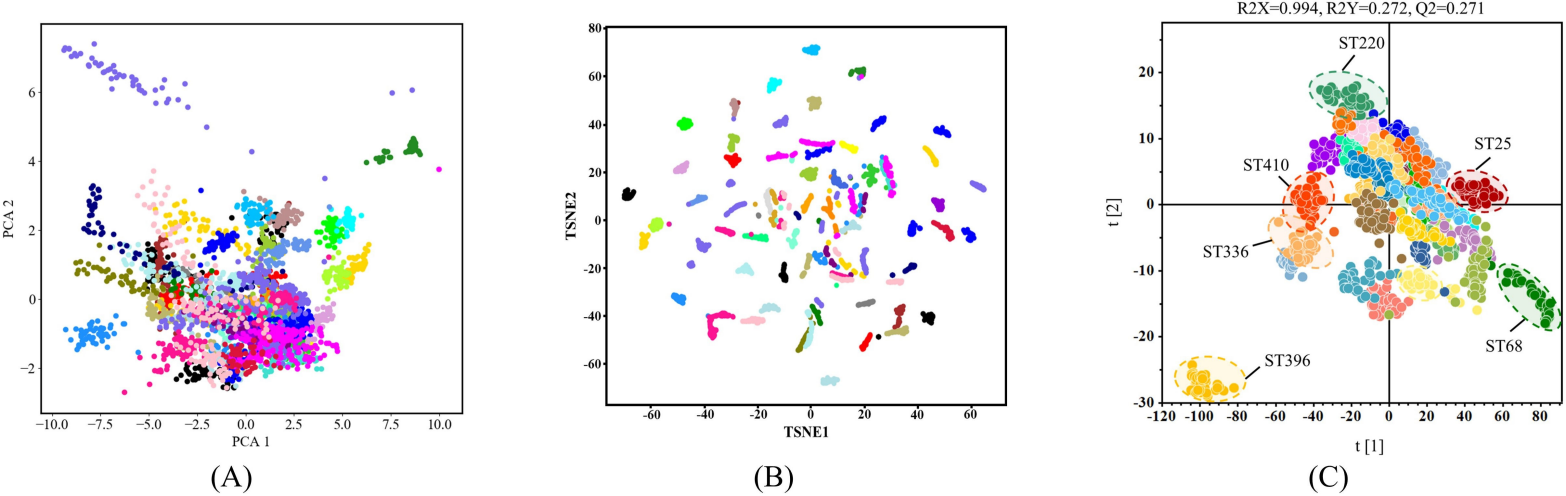
Comparative analysis based on three clustering algorithms (PCA, TSNE, OPLS-DA) in distinguishing 39 STs of *A. baumannii* strains. (**A**) Clustering scatterplot of PCA algorithm. (**B**) Clustering scatterplot of the TSNE algorithm. (**C**) Clustering scatterplot of OPLS-DA algorithm. The overlap of SERS spectral data points for most STs indicates the poor capacities of these clustering algorithms.

Spectral deconvolution provides detailed insights into different STs. However, the rapid identification and classification of distinct STs remain challenging. Clustering analysis can uncover latent patterns among similar spectra. We employed three clustering algorithms to perform dimensionality reduction on the SERS spectra from 39 STs, analyzing their distribution within a two-dimensional space. As shown in the PCA (Fig. 4A) and TSNE results (Fig. 4B), substantial overlap exists among different SERS signal categories, rendering the distinction between various STs difficult. The OPLS-DA results indicate that some STs can be distinguished, though there are still overlaps of STs (Fig. 4C). Moreover, the value 0.271 of Q2 means limited predictive performance for unknown data. Thus, more advanced methodologies are required to distinguish different STs of *A. baumannii* strains.

### Comparison and evaluation of ML algorithms

To address this challenge, eight ML algorithms were deployed to determine the STs of *A. baumannii* strains through the predictive analysis of SERS spectra, and the performance was evaluated using five evaluation metrics. The results in Table 2 revealed that the SVM algorithm performed well in terms of both performance (accuracy = 99.49%) and robustness (fivefold CV = 99.74%). The remaining algorithms, except AdaBoost and QDA, also achieved more than 90% identification accuracy, indicating that these algorithms can serve as effective methods for distinguishing different STs of *A. baumannii* strains. However, AdaBoost has difficulty in fitting a classifier model when processing high-dimensional and multi-category spectral data, and QDA cannot effectively construct a usable covariance matrix for each category, which leads to the poor performance of these two algorithms.

**TABLE 2:** Evaluation of eight machine-learning algorithms for *A. baumannii* STs typing

Algorithms	Accuracy (%)	Precision (%)	Recall (%)	F1 score (%)	Fivefold CV (%)
SVM	99.49	99.49	99.62	99.50	99.74
RF	97.22	97.22	97.56	97.22	98.02
Bagging	96.70	96.70	97.36	96.63	96.55
XGBoost	95.69	95.69	96.37	95.61	94.05
GBoost	91.37	91.37	92.15	91.50	90.52
DT	90.61	90.61	90.91	90.61	87.48
AdaBoost	68.78	68.78	70.32	66.33	65.20
QDA	62.94	62.94	65.48	60.43	60.14

**Fig 5:**
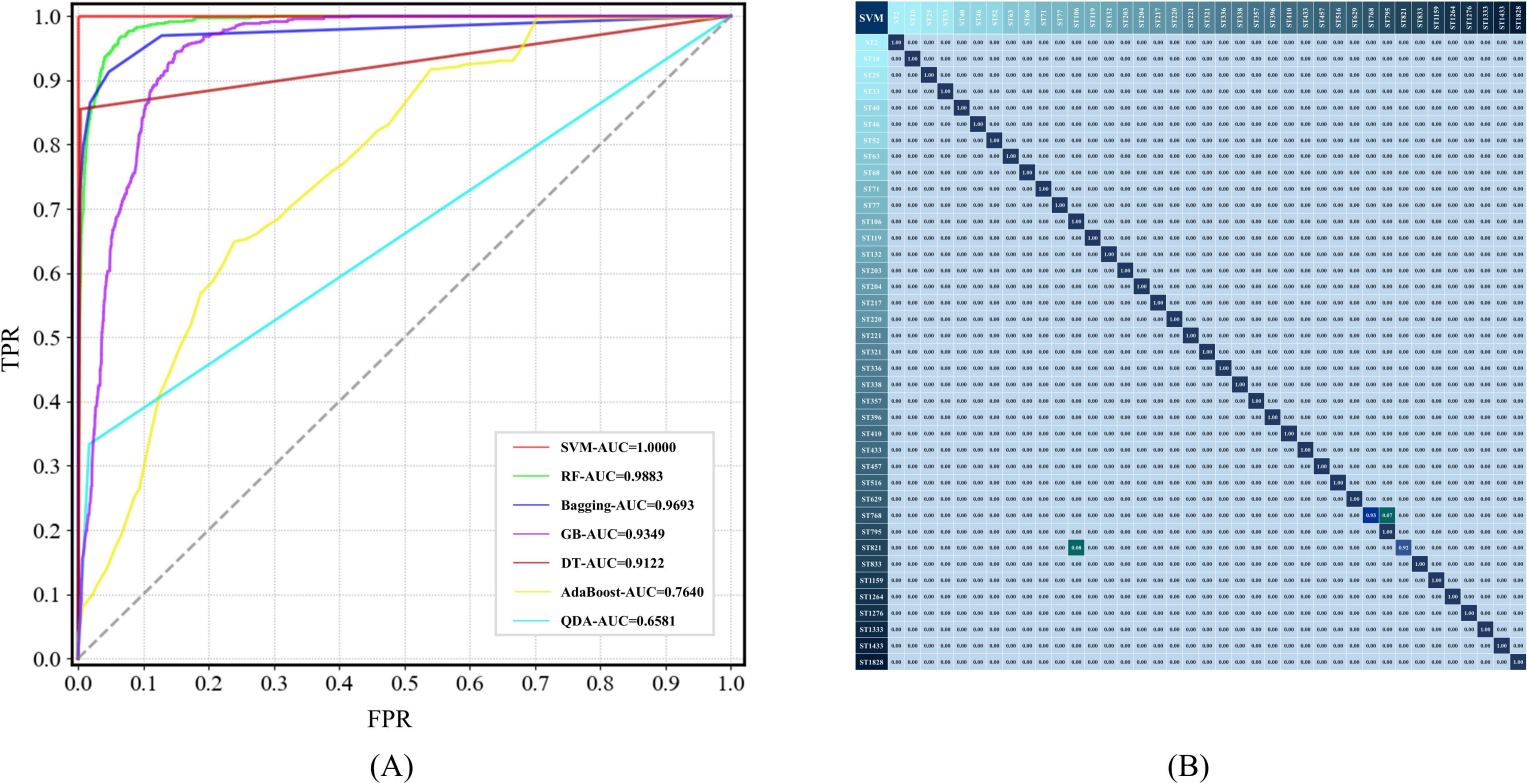
Comparative analysis of the capacities of eight ML models in distinguishing 39 STs of *A. baumannii* strains. (**A**) ROC plots for the eight ML models. (**B**) Confusion matrix of the SVM model.

To corroborate these findings, ROC plots were generated, indicating that the SVM exhibited the highest area under the curve (AUC) value for the area under the ROC curve, reinforcing its superior performance (Fig. 5A). Further evaluation of the SVM through a confusion matrix indicated that the SVM accurately predicted most STs, with misidentification rates of merely 7% and 8% for ST768 and ST821, respectively (Fig. 5B). These findings advocate using the SVM algorithm as the most fitting ML algorithm in this study, as it provides a robust and reliable method for differentiating between various STs.

## DISCUSSION

Prevention of the nosocomial spread of drug-resistant pathogens has emerged as a crucial aspect in hospitals (24). The prompt and accurate identification of microbial species plays a pivotal role in curbing the transmission of infectious agents within healthcare settings (25, 26). The *A. baumannii* infection, known for its ease of transmission and propensity for drug resistance, presents a significant challenge in the realm of nosocomial infection prevention and control. This study introduces two noteworthy innovations. First, SERS can differentiate between *A. baumannii* strains with distinct STs, as evidenced by their distinct Raman spectral feature profiles. Second, we developed and compared eight ML methods based on microbial SERS signals for the rapid identification of bacterial species. The optimal model based on the SVM algorithm was able to quickly fit spectral features within seconds to predict the STs of *A. baumannii*. This approach facilitates the prompt identification of *A. baumannii* variants, aiding in the early detection of nosocomial infections.

The genotypic and phenotypic similarities among various *A. baumannii* strains render their identification complex (27, 28). Traditional methods often demand extensive operational resources in clinical settings. While conventional Raman spectroscopy can identify microorganisms, its sensitivity is frequently inadequate. However, SERS presents a compelling solution. By leveraging the spectroscopic phenomenon of SERS, which involves the adsorption of target molecules onto rough metallic nanomaterial surfaces, the Raman signal of the target is significantly enhanced (29). This addresses the low-sensitivity limitations associated with conventional Raman spectroscopy. SERS has demonstrated promising potential for rapid and accurate clinical identification of pathogens, although its widespread adoption has been limited (30). A major challenge is the difficulty operators face in memorizing the characteristics and differences of numerous strains. To address this, ML algorithm was developed to facilitate the identification of pathogens and their corresponding STs. Notably, previous successful applications of SERS-ML for differentiating between *Shigella* spp. and *Escherichia coli* have been reported (31). In this study, we applied this method for the first time to identify *A. baumannii*, significantly reducing the complexity of its use. These findings underscore the potential of SERS + ML for clinical applications.

The intrinsic capabilities of statistical and machine-learning models directly influence the efficiency and accuracy of microbial identification (32). Three cluster analysis algorithms and eight different ML algorithms were applied to address this issue. Currently, only the SVM algorithm fulfills our criteria. However, this approach has not yet achieved 100% accuracy, as some STs are still incorrectly identified. Furthermore, as the sample size expands, the recognition error rate of SVM may increase accordingly, which is a topic for future research. In addition, RF, Bagging, XGBoost, and GBoost exhibited recognition rates above 90%, yet they did not match the SVM’s performance. Future efforts will involve considering multiple algorithms simultaneously to enhance recognition accuracy. This would entail using various ML algorithms to analyze the same sample and accepting the result only if all these algorithms concur. However, due to time, budget, and technology constraints, not all algorithms could be tested in the current study, marking another direction for future enhancement of the recognition algorithms (33). Overall, while SVMs have met the current demands for determining *A. baumannii* STs, further research is necessary to improve the recognition accuracy and refine the algorithm selection.

This study is subject to certain limitations. First, this was a single-center study, and the data in the sample pool only represented the STs of *A. baumannii* identified within Guangdong Provincial People’s Hospital, China. Consequently, the model cannot recognize STs out of the scope of this study. Furthermore, the algorithms used in this study do not achieve 100% recognition accuracy, highlighting the need to further optimize the model through increasing sample sizes or explore additional more advanced algorithms.

### Conclusions

This study successfully developed a diagnostic method capable of accurately identifying the STs of clinically isolated *A. baumannii* strains based on the SERS spectra. This rapid and straightforward identification process is important for effectively preventing and controlling nosocomial transmission of *A. baumannii*. The predictive model will be expanded through multicenter studies to enhance its applicability and accuracy in the future.

## Data Availability

The raw data supporting the conclusions of this article will be made available by the authors without undue reservation. No data sets were generated or analyzed during the current study.

## SUPPLEMENTAL MATERIAL

Fig. S1 spectrum.01425-24-s0001.docx Dot plot of bacterial ST typing characteristic peaks distribution. The dot plot displays the combinations and intensity distributions of different STs type of characteristic peaks. By comparing the differences between these characteristic peaks, it can aid in the accurate identification of specific STs.

ASM does not own the copyrights to Supplemental Material that may be linked to, or accessed through, an article. The authors have granted ASM a non-exclusive, world-wide license to publish the Supplemental Material files. Please contact the corresponding author directly for reuse.
