# Supplementary material for: Comparison of Raman spectroscopy with mass spectrometry for sequence typing of Acinetobacter baumannii strains: a single-center study
Source: Microbiol Spectr. 2025 Feb 5;13(3):e01425-24. doi: 10.1128/spectrum.01425-24 (PMC11878063; doi:10.1128/spectrum.01425-24)
Supplement: Fig. S1 — Dot plot of bacterial ST typing characteristic peaks distribution. The dot plot displays the combinations and intensity distributions of different STs type of characteristic peaks. By comparing the differences between these characteristic peaks, it can aid in the accurate identification of specific STs. [file spectrum.01425-24-s0001.docx]

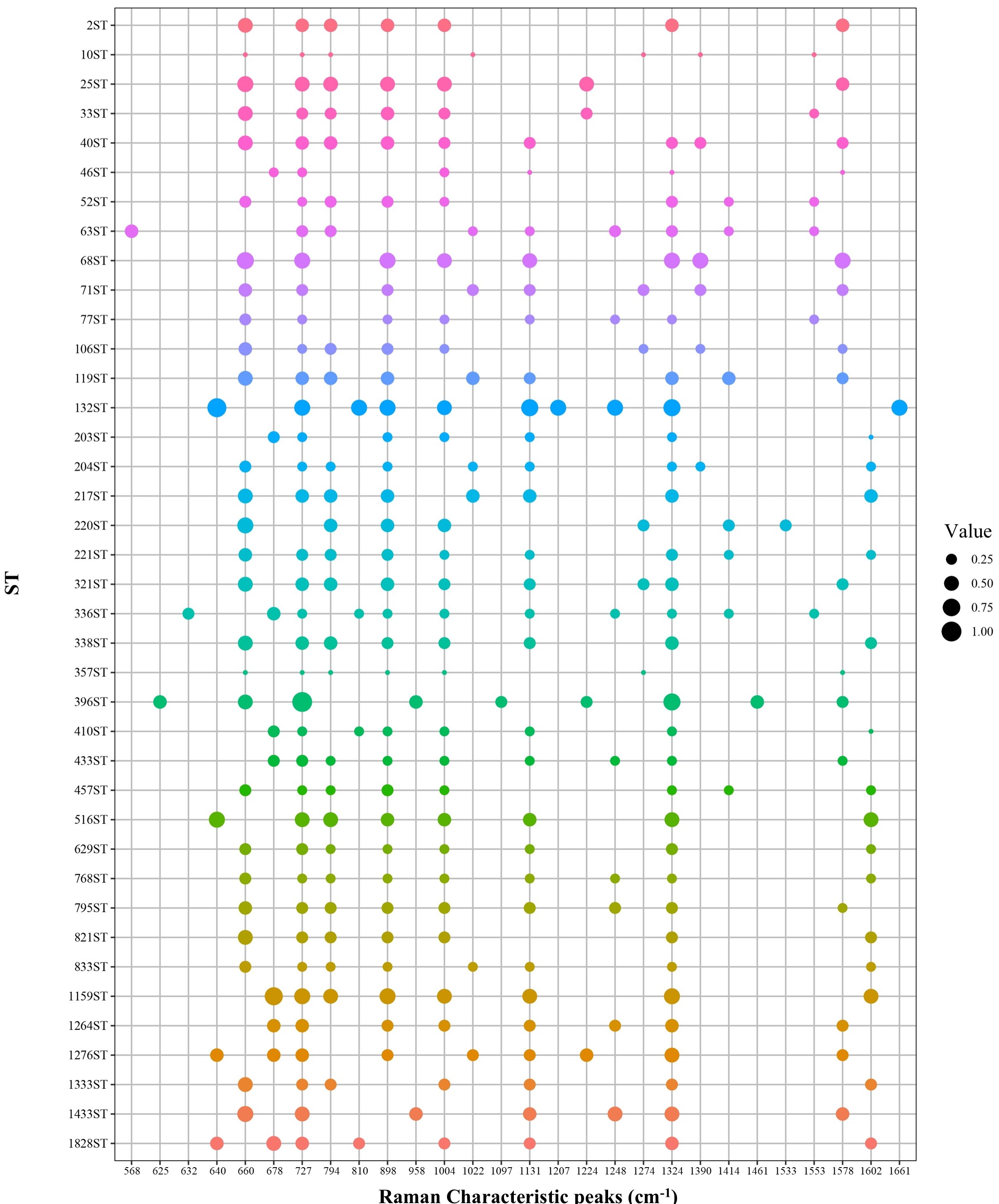


**Supplementary Figure S1** Dot plot of bacterial ST typing characteristic peaks distribution. The dot plot displays the combinations and intensity distributions of different STs type of characteristic peaks. By comparing the differences between these characteristic peaks, it can aid in the accurate identification of specific STs.
